# From inflammation to gastric cancer – the importance of Hedgehog/GLI signaling in *Helicobacter pylori*-induced chronic inflammatory and neoplastic diseases

**DOI:** 10.1186/s12964-017-0171-4

**Published:** 2017-04-20

**Authors:** Silja Wessler, Linda M. Krisch, Dominik P. Elmer, Fritz Aberger

**Affiliations:** 1 Division of Microbiology, Cancer Cluster Salzburg, Department of Molecular Biology, Paris-Lodron University of Salzburg, Billroth Strasse 11, A-5020 Salzburg, Austria; 2 Division of Molecular Tumor Biology, Cancer Cluster Salzburg, Department of Molecular Biology, Paris-Lodron University of Salzburg, Hellbrunner Strasse 34, A-5020 Salzburg, Austria

**Keywords:** *Helicobacter pylori*, Gastric cancer, Hedgehog/GLI signaling, Tumor microenvironment

## Abstract

Infections with the human pathogen *Helicobacter pylori* (*H. pylori*) are closely associated with the development of inflammatory disorders and neoplastic transformation of the gastric epithelium. Drastic changes in the micromilieu involve a complex network of *H. pylori*-regulated signal transduction pathways leading to the release of proinflammatory cytokines, gut hormones and a wide range of signaling molecules. Besides controlling embryonic development, the Hedgehog/GLI signaling pathway also plays important roles in epithelial proliferation, differentiation, and regeneration of the gastric physiology, but also in the induction and progression of inflammation and neoplastic transformation in *H. pylori* infections. Here, we summarize recent findings of *H. pylori*-associated Hedgehog/GLI signaling in gastric homeostasis, malignant development and the modulation of the gastric tumor microenvironment.

## Background

Although the incidence of gastric cancer steadily declined in the last 20 years, stomach cancer is still the second leading cause for cancer-related deaths worldwide [1]. As the major causative agent for gastric cancer, the human bacterial pathogen *Helicobacter pylori* (*H. pylori*) has been identified, which is responsible for more than 70% of gastric adenocarcinomas (non-cardia gastric cancers) and also for other gastric disorders including chronic gastritis, ulceration of the stomach and duodenum, and lymphomas of the mucosa-associated lymphoid tissue (MALT) system [2, 3]. According to the strong association between infections with *H. pylori* and neoplastic transformations in the human stomach, *H. pylori* has been classified as a class-I carcinogen, representing the strongest known risk factor for gastric cancer [4].

Gastric cancer can be histologically differentiated between diffuse or intestinal types and both are linked to chronic *H. pylori* infections in humans. The pathogenesis of the diffuse-type carcinoma is less well understood, but has been frequently associated with the loss of expression of the cell adhesion molecule and tumor suppressor E-cadherin (CDH1). Loss of E-cadherin function is often the consequence of *cdh1* germline mutations and could also be linked to sporadic mutations or promoter hypermethylation. Tumor cells exhibiting CDH1 malfunction and subsequently loss of intercellular adhesions tend to invade adjacent tissues and are considered as more aggressive compared to tumor cells of the intestinal type [5, 6]. Gastric cancer of the intestinal type typically involves a series of sequential processes, which are strongly linked to *H. pylori* infections. According to the Correa’s cascade, chronic active inflammation in response to persistent *H. pylori* infection represents the initial phase in carcinogenesis followed by chronic atrophic gastritis, intestinal metaplasia, dysplasia, and finally invasive carcinoma [6]. During gastric carcinogenesis, genetic abnormalities accumulate and may involve mutations in the *APC*, *TP53*, and *KRAS* genes, but also hypermethylation and microsatellites were detected [7, 8].

**Fig. 1:**
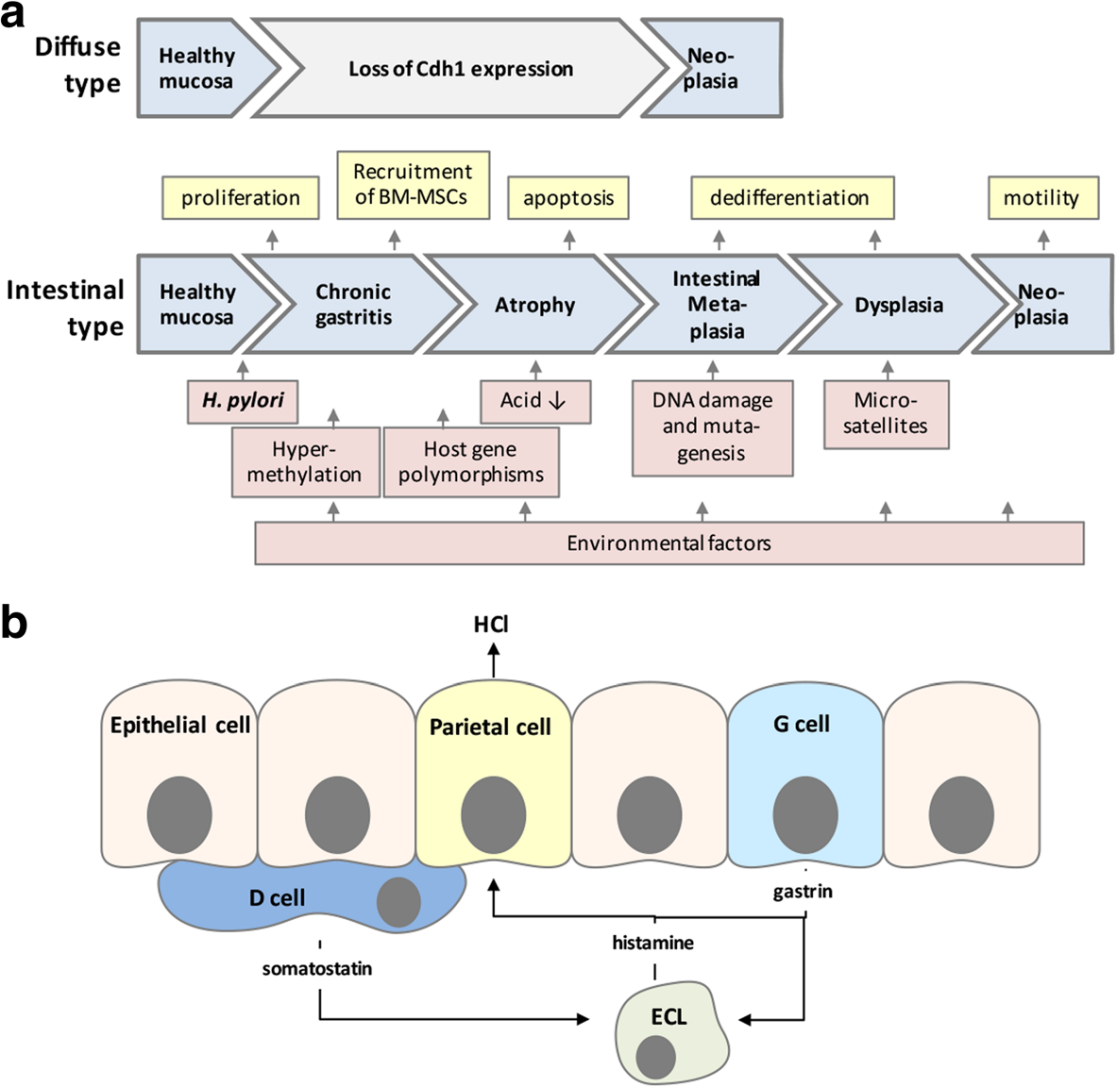
Model of the gastric physiology and cancer development. **a** Infection with *H. pylori* has been strongly associated with the development of the diffuse type and the intestinal type of gastric cancer. The diffuse type is often accompanied by the loss of E-cadherin (CDH1) expression. The development of the intestinal type of gastric cancer is associated with chronic gastritis, atrophy, and intestinal metaplasia as precursors of dysplastic changes. Mutations, hypermethylation, and microsatellites, but also environmental factors are implicated in the carcinogenic process. In this model, chronic active inflammation represents the initial phase in carcinogenesis via alterations of epithelial apoptosis, cell proliferation, recruitment of BM-MSCs, dedifferentiation processes and induced invasive growth of neoplastic cells. **b** The gastric physiology is established by the coordinated action of paracrine factors and hormones. The epithelium contains parietal cells, D cells, G cells and circulating enterochromaffin-like (ECL) cells. The release of gastric acid by parietal cells is stimulated by ECL-secreted histamine and gastrin expressed by G cells. D cells produce the negative regulator somatostatin, which blocks acid secretion via direct effects on parietal cells and through the inhibition of histamine and gastrin release

Normally, *H. pylori* infection is acquired in childhood, and persists for the patient’s lifetime if not treated with antibiotics. Although infections with *H. pylori* are prevalent, only approximately 1–3% of the patients develop gastric cancer [3]. The clinical outcome strongly depends on the crosstalk between strain-specific bacterial virulence factors, genetic predispositions of the host, alterations of the stem cell niche, microbiota and environmental influences. In this context, implications of gene polymorphisms have been described including interleukins and antagonistic receptors such as *IL1B*, *IL10*, *IL1RN* and *TNF-alpha* [9, 10]. Environmental factors include smoking, high salt consumption, processed meat or alcohol as possible risk factors for gastric cancer (Fig. 1a). In contrast, consumption of fresh fruits and vegetables has been associated with reduced cancer risk. The major bacterial determinant in the risk of developing gastric cancer is represented by the cytotoxin-associated gene pathogenicity island (*cag*PAI). The *cag*PAI is a 40 kB DNA insertion element consisting of 27-31 genes that encode proteins important for the structure and function of a highly-specialized type IV secretion system (T4SS) [11]. The T4SS translocates the only known effector protein cytotoxin-associated gene A (CagA) into the cytoplasm of infected gastric epithelial and immune host cells where it is tyrosine phosphorylated by non-receptor tyrosine kinases of the Src and Abl kinase families [12–14] and derails cancer-associated signal transduction pathways [15, 16]. In fact, infections with CagA-positive *H. pylori* strains have been strongly correlated with the development of severe inflammatory responses and subsequently gastric cancer [17]. It has been suggested that in comparison to *cagA*-negative isolates, *H. pylori* strains expressing CagA increase the risk of distal gastric adenocarcinoma twofold [18]. Using in vivo animal models, CagA translocation has been suggested to play an important role in the induction of gastric cancer [19, 20]. Transgenic mice systemically expressing CagA underlined this observation through the finding that CagA increased gastric epithelial cell proliferation rates and carcinomas [21]. Besides CagA, the expression of additional factors was described as further important bacterial determinant in the development of gastric cancer, such as vacuolating cytotoxin A (VacA) [22, 23], adhesion factors as blood group antigen-binding adhesin (BabA) [24] and sialic acid-binding adhesin (SabA) [25].

### *H. pylori* induced gastric cancer and the tumor microenvironment

The mechanism of how *H. pylori* can induce gastric cancer is not well understood. *H. pylori* induces a plethora of different signal transduction processes that trigger a complex multi-step process leading to inflammation and carcinogenesis [26–29]. Normally, these pathways critically control cellular responses such as proliferation, apoptosis, epithelial dedifferentiation and motility, thereby regulating tissue homeostasis (Fig. 1a). So far, most studies of *H. pylori* induced cancer have focused on specific cell types, although the interplay between different cell types ranging from gastric epithelial cells, glands, immune cells, to stem cells is crucially important for the development and progression of *H. pylori*-associated carcinogenesis [30–32].

*H. pylori* associated gastric cancer is characterized by a chronic inflammatory phenotype, where the contribution and interaction of bacterial virulence factors and the host immune system account for oncogenic transformation (for review see [30] and references therein). This becomes evident at the molecular as well as cellular level. For instance, *H. pylori* has been reported to activate the key inflammatory regulator nuclear factor kappa B (NF-κB), resulting in the activation and enhancement of cytokine signaling including IL-8 and TNF-alpha [33–38]. Further, IL11 mediated activation of STAT3, an important regulator of inflammation and driver of carcinogenesis, is a hallmark of about 50 percent of gastric cancers and has been shown to contribute to tumor growth within an inflammatory setting [39, 40]. At the cellular level, myeloid and lymphocytic cells frequently infiltrate malignant lesions. Tumor-associated macrophages (TAM) promote malignant progression and the degree of TAM-infiltration induced by a variety of chemoattractant factors correlates with tumor progression and clinical disease stage [41–43]. Also, the number of immunosuppressive regulatory T-cells (Tregs) is enhanced in tumor-draining lymph nodes and peripheral blood of gastric cancer patients and the number of Tregs inversely correlates with the survival of patients [44–49]. Besides cells of the innate and adaptive immune system, the tumor microenvironment is to a large degree made up of cancer-associated fibroblasts (CAF) that develop in response to the interplay of cancer cells with their stromal environment. CAF support cancer growth and progression by producing pro-tumorigenic and -metastatic factors including pro-angiogenic signals [50–53]. Thus, a detailed understanding of oncogenic signaling pathways within the tumor and stromal compartments, particularly also in inflammatory and immunosuppressive cell types is needed to guide the design of novel combination therapies that may involve strategies blocking both immunosuppressive and pro-tumorigenic inflammatory signals in the tumor microenvironment together with targeted inhibition of oncogenic driver cues in gastric cancer cells.

### Gastric physiology and Hedgehog/GLI signaling in gastric cancer

Dependent on the region in the human stomach, the gastric epithelial lining forms foveolae consisting of different types of cells and glands, including mucous, endocrine, and undifferentiated cells (Fig. 1b), which coordinate the complex gastric physiology by a balanced micromilieu. Embedded within undifferentiated epithelial cells, D cells, G cells and circulating enterochromaffin-like (ECL) cells release regulatory molecules controlling the production of gastric acid by parietal cells [54, 55]. Histamine is released from ECL cells, the hormone gastrin is released by G cells, and the hormone somatostatin is secreted by D cells. In a paracrine manner, histamine stimulates parietal cells to produce gastric acid. Gastrin is involved in acid secretion, stimulating histamine release from ECL cells. As a negative regulator, somatostatin release is stimulated when the pH in the stomach is too low. Then it blocks acid secretion via direct effects on parietal cells, but also through the inhibition of histamine and gastrin release [54, 55] (Fig. 1b). This sensitive balance of intercellular communication can be crucially interrupted by infections with *H. pylori* through manifold mechanisms [56, 57]. As an additional important part of changes in the gastric tumor microenvironment, *H. pylori* stimulates a wide range of proinflammatory mediators employing a highly complex network of a wide range of diverse signaling pathways [16, 58, 59]. In fact, relatively little is known about the detailed molecular processes and signals operating during the early and later stages of gastric cancer in response to *H. pylori* infection and chronic inflammation. In recent years, several oncogenic pathways including the wingless-type MMTV integration site family (Wnt)/beta catenin, NF-κB and Hedgehog/GLI (HH/GLI) signaling pathway have been implemented in the complex network of diverse molecular mechanisms leading to gastric cancer [60]. The implication of HH/GLI signaling in gastric cancer has, therefore, opened the possibility of HH/GLI targeting as a novel therapeutic approach.

The HH/GLI pathway, first discovered in a mutagenesis screen for embryonic patterning mutants of the fruit fly [61], is a crucial developmental regulatory signal that has been highly conserved throughout various phyla. During the past years, HH/GLI signaling has attracted substantial interest by tumor biologist and oncologist because of its widespread hyperactivation and oncogenic activity in a variety of human malignancies. In fact, HH/GLI signaling and its target genes control the major hallmarks of cancer and cancer stem cells including proliferation, survival, metastasis, angiogenesis and self-renewal, making this signaling pathway a promising target for therapies [62–66].

**Fig. 2:**
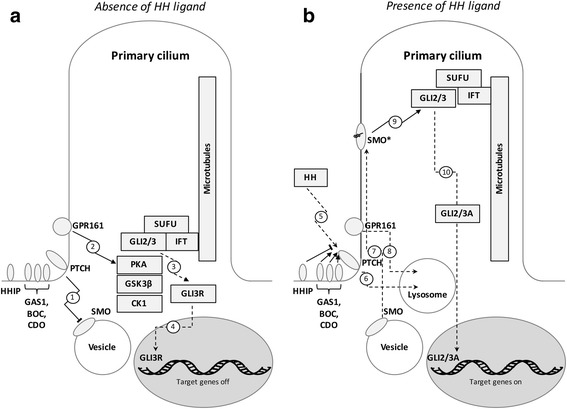
A simplified schematic depiction of the canonical hedgehog (HH) signal transduction pathway within the primary cilium. **a** During the absence of the HH ligand the pathway is continuously repressed by PTCH and GPR161 located at the base of the primary cilium. In its unliganded state the HH receptor PTCH prevents SMO, the crucial GLI activator, from entering the plasma membrane of the primary cilium and thereby from executing its effector function (1). Furthermore, GPR161 increases cAMP levels, promoting the phosphorylation of the GLI transcription factors, which are sequestered in a repressive complex with SUFU and IFT proteins at the base of the primary cilium, by PKA and subsequently by GSK3β and CK1 (2). This phosphorylation leads to partial proteasomal degradation of GLI2 and GLI3 and repressor formation, predominantly GLI3R (3). Thereupon, GLI3R enters the nucleus and represses target gene transcription (4). **b** The binding of the HH ligand to its receptor PTCH is promoted by GAS1, BOC and CDO, whereas HHIP competes with PTCH for ligand binding (5). When HH binds to PTCH the repression of the pathway is relieved by internalization and lysosomal degradation of the receptor-ligand-complex (6). This allows SMO to enter the primary cilium (7) and to be activated by cholesterol, which triggers a conformational change (indicated by the schematic cholesterol structure in black and the asterisk (SMO*)). GPR161 whereas is removed from the plasma membrane (8). When SMO* and the SUFU-GLI complex co-localize at the tip of the primary cilium, upon the directed transport via IFT proteins along the microtubules, the GLI transcription factors are activated by SMO* and dissociate from the complex (9). The full-length activator forms of GLI2 and GLI3 enter the nucleus and drive target gene transcription (10)

The HH/GLI pathway is a highly complex signal transduction process involving numerous regulatory factors and control mechanisms located in different cellular compartments. In a nutshell, during the off-state canonical HH/GLI signaling is actively repressed via the unliganded, twelve-pass transmembrane HH receptor patched (PTCH) (Fig. 2a). PTCH prohibits the pathway activator Smoothened (SMO), a G-protein coupled receptor-like protein, from entering the primary cilium. In this repressed state, the negative pathway regulator suppressor of fused (SUFU) sequesters the first-line effector proteins, glioblastoma-associated-protein 2 and 3 (GLI2, GLI3) in the cytoplasm at the base of the primary cilium. The formation of the SUFU-GLI protein complex allows the sequential phosphorylation of the GLI proteins by protein kinase A (PKA), glycogen synthase kinase 3 beta (GSK3β) and casein kinase 1 (CK1) [67, 68]. Upon phosphorylation, GLI2 and GLI3 are ubiquitinylated and partially degraded by the proteasome located at the base of the primary cilium [69]. While partial degradation of GLI2 is rather inefficient, GLI3 is known to abundantly form stable transcriptional repressors upon proteolytic removal of the C-terminal portion harboring the transactivation domain [70, 71]. Thus, proteolytic processing yields a GLI repressor form (GLIR) that prevents and shuts off HH target gene expression (for more extensive reviews on HH/GLI signaling see [72–78]).

The canonical HH/GLI pathway is activated via binding of the HH ligand to the receptor PTCH (Fig. 2b). Ligand binding abrogates the repressive action of PTCH, leading to the internalization of the receptor-ligand complex and its subsequent degradation in lysosomes. HH ligand binding is influenced by the presence of distinct co-receptors: growth arrest specific 1 (GAS1), cell adhesion molecule-related/down-regulated by oncogenes (CDO) and brother of CDO (BOC) support the binding of HH to PTCH, while hedgehog interacting protein (HHIP) competes with PTCH for the HH ligand [79–82]. The removal of PTCH triggers the entry of SMO into and the exit of GPR161 from the primary cilium. SMO is either shifted laterally within the plasma membrane or enters the cilium from intracellular vesicles [83]. There is evidence that the removal of GPR161 is sufficient to prevent GLIR formation, most likely because of a reduced PKA activity [84–86]. GLI activation, however, is crucially dependent on the activation and the correct localization of SMO. Although the specific signal, which activates and represses SMO in response to HH, has not yet been identified, recent work by the Rohatgi group has shed light on the regulatory role of conformational changes of SMO for signal transduction. These studies revealed that cholesterol binding to the extracellular SMO domains stabilizes a conformation that promotes responsiveness towards activating stimuli [87, 88].

When the pathway is activated the GLI-SUFU complex is transported from the base to the tip of the primary cilium. It has been shown that only upon the co-localization of active SMO and GLIs at the tip of the primary cilium, full-length GLI2 and GLI3 are released from SUFU. The full-length GLIs then translocate into the nucleus to activate target gene transcription [69, 89, 90]. Upon GLI activation, positive as well as negative feedback loops are elicited to balance the strength and the duration of pathway activation. *GLI1* encodes a second-line but critical pathway amplifier that is directly induced by GLI2 [91, 92]. GLI1 potently amplifies HH/GLI signaling by activating and/or enhancing the expression of a battery of HH target genes. Fine-tuning of the response to GLI activity further depends on interactions with co-factors, post-translational modifications including phosphorylation and acetylation as well as on the differential stability and degradation of the GLIs [93–98]. In addition, different target genes display different sensitivities towards GLI activator (GLIA) and GLIR levels as a consequence of GLI binding site variations with distinct affinities, adding another regulatory layer for the precise determination of the response to the so-called GLI-code [99–102].

Further, there is a steadily increasing list of mechanisms accounting for SMO-independent regulation of GLI activity and expression. Of note, these non-canonical HH/GLI signals have been repeatedly reported in cancer cells, integrating the HH/GLI pathway in the complex web of oncogenic signals but also accounting for resistance to clinical inhibitors targeting SMO, which has become a major challenge for the use of Hedgehog pathway inhibitors in oncology [103–110] (for comprehensive reviews see [102, 111, 112]).

### HH/GLI targeting as therapeutic option in gastric cancer – challenges and considerations

Despite substantial efforts of biotech and pharmaceutical companies to develop efficient HH pathway inhibitors, the clinical success of anti-HH therapies has mainly been limited to non-melanoma skin and brain cancers, while other clinical trials using HH/GLI inhibitors for the treatment of solid cancers with high medical need yielded largely disappointing results [113–118]. Although these failed trials were based on sound preclinical evidence supporting a key role of HH/GLI signaling in malignant progression of various cancer entities [119–123], the unforeseeable complexity of HH/GLI signal regulation within the tumor and its microenvironment as well as the frequent development of *a priori* and/or acquired drug resistance have recently challenged the concept of HH/GLI targeting in oncology [124, 125]. We outline two examples – HH/GLI signaling in pancreatic and colorectal cancer - to emphasize the strict need for a very careful and comprehensive analysis of the oncogenicity of the HH/GLI pathway within the complex interplay of cancer cells with their microenvironment and the immune system, in order to develop multi-modal therapeutic protocols that may be successful in the future treatment of gastric cancer.

First evidence based on in vitro and xenograft models suggested a crucial tumor-cell autonomous role of canonical HH/GLI signaling in pancreatic cancer [119, 126]. However, this concept has recently been challenged by findings showing in vivo activation of HH/GLI signaling in the stromal rather than tumor cell compartment. Strikingly, inhibition of HH/GLI signaling in the tumor stroma of pancreatic cancer led to enhanced tumor growth rather than a therapeutic effect, reflecting the discouraging outcome of anti-HH trials in pancreatic cancer patients [127–129]. By contrast, non-canonical activation of the GLI transcription factors mediating HH/GLI signaling in the nucleus of pancreatic cancer cells is essential for tumor initiation and disease progression [130, 131]. This suggested that direct targeting of oncogenic GLI proteins - while also maintaining the protective effect of the stromal compartment - may prove a successful therapeutic strategy within a multi-modal combination treatment protocol.

Similarly, the initial enthusiasm about HH targeting for the treatment of colorectal cancer faded rapidly, when clinical trials with HH antagonists did not show any significant therapeutic benefit. This may to some extent be due to the fact that most preclinical models used for studying the oncogenic effect of HH/GLI signaling did not take into account the cellular and molecular complexity of the tumor microenvironment and the vivid cross-talk between tumor cells, the tumor stroma and the immune system. Like in pancreatic cancer, it has recently been shown that canonical HH/GLI signaling in colon cancer is strongly activated in the stromal rather than the tumor cell compartment providing a cancer-protective activity. Intriguingly, HH/GLI signaling in the stromal compartment of mouse colon cancer models reduces tumor development by modifying BMP signaling in colon cancer cells and by dampening inflammatory signaling in colitis-associated cancer models [132, 133]. Given the distinct functions of HH/GLI signaling within the heterogeneous cellular context of the tumor and its microenvironment, a precise understanding of HH/GLI signaling in the context of gastric cancer is mandatory for the future evaluation of the therapeutic potential of HH/GLI targeting.

### Hedgehog signaling as a crucial mediator in gastric physiology and disease

The role of the HH/GLI signaling pathway in gastric homeostasis has been established in several recent studies (for reviews see [134–136]). The expression of the hedgehog family member sonic hedgehog (SHH) is required to shape the mucosal layer but has to be tightly controlled during the development of the gastric glandular epithelium [135, 137]. Furthermore, SHH expression appears to be crucial for gastric tissue repair [138] and for the maintenance of the functional morphology and the regulation of secretory functions of gastric glands in adult mice [134]. There is evidence that SHH production and reception by parietal cells is required to maintain the acid and gastrin secretion in the stomach at physiological levels. Furthermore, the SHH concentration gradient established by the parietal cells, located in the central region of the gland seems to support the differentiation of mucous neck to zymogenic cells. At the same time, high concentrations of the secreted SHH ligand seem to restrict the proliferation of surface pit cells [139, 140].

**Fig. 3:**
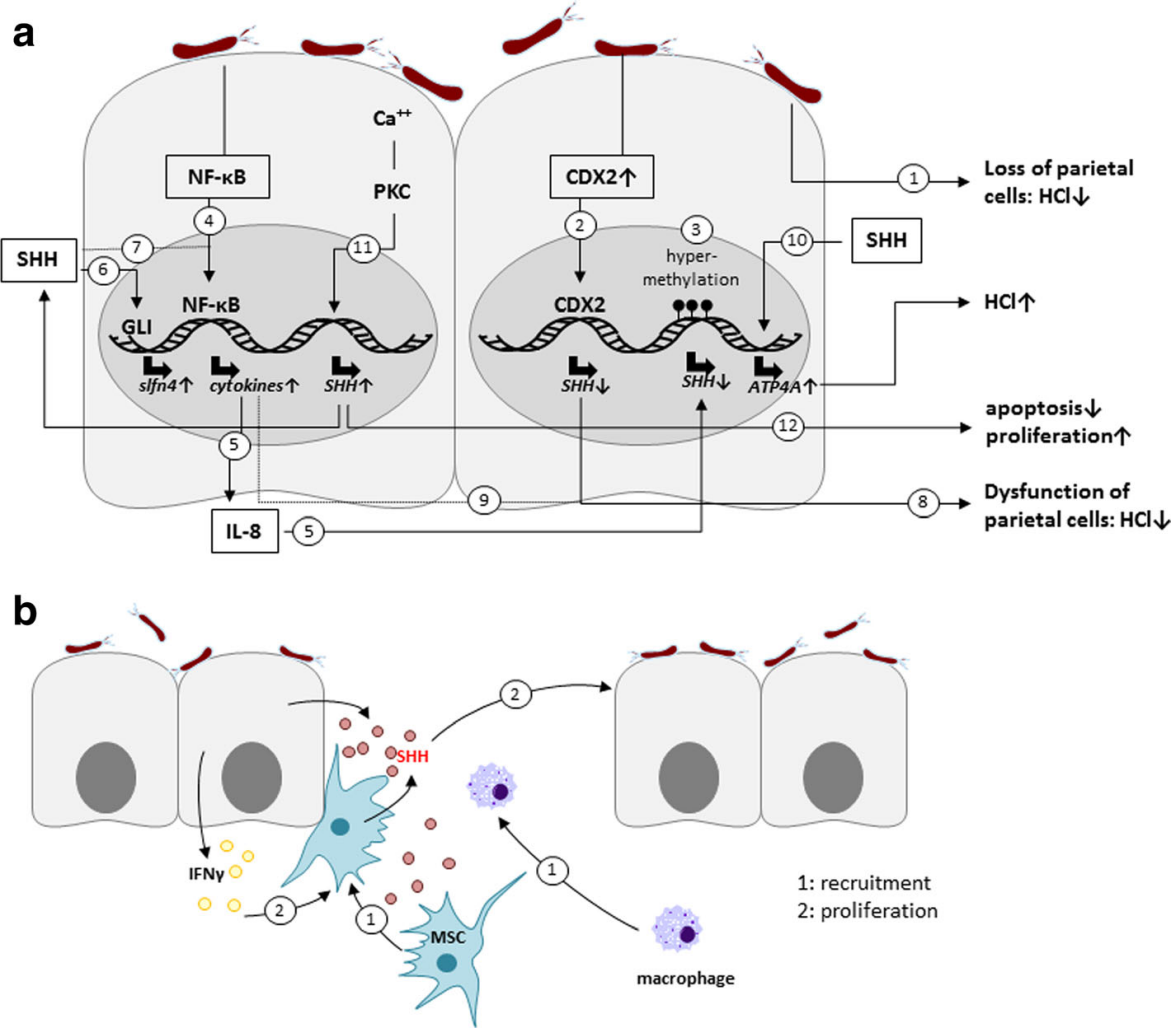
Model of *H. pylori* interference with the SHH signaling pathway. **a** Infection with *H. pylori* induces a loss of SHH-expressing parietal cells (1). Loss of SHH might involve *H. pylori*-induced CDX2 expression, which then binds the SHH gene promoter leading to SHH downregulation (2) and to a dysfunction of parietal cells (8). Downregulation of SHH has also been associated with hypermethylation of the promoters of hedgehog signaling genes (3). The negative effect of *H. pylori* on SHH expression involves NF-κB activity (4) that induces the expression of cytokines to reduce SHH gene expression (5). On the other hand, SHH can be upregulated in response to calcium and PKC activity (11), which leads to the activation of GLI expression to enhance *schlafen-4* (6) or increases H^+^/K^+^-ATPase gene expression (10). A positive effect of SHH on cytokine expression was observed (7), which could contribute to the NF-κB responses. *H. pylori*-induced proinflammatory cytokines further the dysfunction of parietal cells to inhibit gastric acid production (9), while an increased SHH expression promotes anti-apoptotic effect and proliferation (12). **b** *H. pylori*-increased SHH can function as a chemoattractant for macrophages and BM-MSCs. IFNγ-induced MSC proliferation required SHH secretion via an autocrine regulatory mechanism and appears to be involved in the repopulation of the inflamed tissue

As SHH plays a crucially important role in cellular differentiation and gastric tissue homeostasis [141], epithelial cell differentiation in *H. pylori*-associated gastritis in the Mongolian gerbil model has been investigated. Persistent infection with *H. pylori* induced inflammation of the antrum and corpus of the stomach, which was accompanied by a clear loss of SHH expression in parietal cells and mucous neck cells of the gastric fundic glands as monitored by quantitative real-time (RT)-PCR, *in situ* hybridization, immunoblotting and immunohistochemistry. This phenotype was associated with the loss of parietal cells and disturbed fundic gland cell differentiation [142] (Fig. 3a). A similar observation was made in human patients underlining a correlation between *H. pylori* infections and the HH signaling components SHH, SMO and GLI2 [143]. In comparison to healthy mucosa, expression of the intestine-specific transcription factor caudal type homeobox 2 (CDX2) negatively correlated with SHH expression in the corpus lesser curve of gastric cancer patients indicating that *H. pylori* might employ SHH expression in gastric atrophy and intestinal metaplasia during the development and/or progression of gastric carcinogenesis [144] (Fig. 3a). CDX2 exhibits an important role in the development and maintenance of the intestinal epithelium, but is frequently found in gastric cancer with controversially discussed functions. The expression of CDX2 in transgenic mouse models transformed the gastric mucosa into intestinal metaplastic mucosa and triggered gastric cancer [145, 146], but in other studies CDX2 expression correlated with a better prognosis [147, 148]. Interestingly, *H. pylori* eradication led to an increase in SHH expression in Mongolian gerbils [149] and in the human corpus, where it mediated a decrease in CDX2 expression in the corpus lesser curve [150–152]. Although eradication of *H. pylori* mediated an increase in SHH expression and its downstream regulators, the beneficial effect was not observed in patients with high risk of gastric cancer [153]. Therefore, it was proposed that prevention of cancer might be improved through *H. pylori* eradication prior to the development of atrophic gastritis with intestinal metaplasia [153]. These observations also point to a functional role of SHH re-expression in the gastric epithelial regeneration. Notably, mice with a parietal cell-specific deletion of *Shh* (*PC-Shh* ^*KO*^) showed a delayed wound healing [154], suggesting that SHH re-expression after *H. pylori* eradication contributes to epithelial regeneration.

### *H. pylori* can directly control SHH expression

The loss of parietal cells associated with the loss of SHH expression during *H. pylori*-mediated atrophy suggests an indirect regulatory effect of *H. pylori* on HH activity. However, *H. pylori* may exert strategies directly targeting SHH expression. A comprehensive profiling of DNA methylation of a well-characterized series of primary gastric cancers was performed and 147 genes were identified exhibiting significantly changed methylation patterns in tumor and matched tumor-adjacent gastric tissue. Among these genes, *hoxA5* and hedgehog signaling molecules (WNT2, WNT5A, SMO, HHIP, GLI3, BMP6) were found [155], suggesting that the loss of hedgehog signaling proteins is also the consequence of epigenetic processes (Fig. 3a). However, the reciprocal expression of SHH and CDX2 in intestinal metaplasia could not be explained by methylation [156]. SHH was drastically down-regulated in a CDX2 transgenic mouse model, which was not mediated by *SHH* promoter hypermethylation. In fact, the authors observed that CDX2 directly binds the TATA box in the *SHH* promoter leading to a down-regulation of the SHH expression, suggesting a direct connection between SHH and CDX2 expression. These observations were confirmed in cultured AGS, MKN45, and MKN74 cells transfected with CDX2-expressing plasmids, in which SHH expression was clearly decreased [156]. Although a cell-type-specific induction of CDX2 expression has been shown in *H. pylori*-infected gastric epithelial cell lines [157, 158], this observation has not yet been correlated with SHH gene regulation.

In insulin-gastrin (InsGas) mice that overexpress pancreatic gastrin to study gastric cancer, SHH, GLI1, but not GLI3 expression in pre-metaplastic lesions of non-infected mice was considerably reduced compared to normal adjacent glands, but partially re-expressed in *H. felis*-induced gastric metaplasia. It was suggested that *H. felis*-activated NF-κB and subsequently IL-8 secretion may be involved in this pathway [159] (Fig. 3a). This indicates that *H. felis* could regulate SHH signaling through the loss of SHH expressing cell types. Similar effects were found in transgenic GLI1-deficient mice infected with *H. felis*, which were largely resistant to the development of gastric metaplasia and infiltration by inflammatory cells [160]. GLI1 deletion blocked Th1 and Th2 cytokines, but not a Th17 response. As a target gene of GLI1, *schlafen-4* was identified in microarray analyses (Fig. 3a), which was induced in wildtype mice, but not in the *Gli1*-deficient mice [160]. In addition, SHH has been shown to positively regulate cytokine expression during *H. pylori* infection [161]. Comparing WT and *PC-Shh* ^*KO*^ mice, an increase of *Il-12*, *Il-1β*, *Il-10*, *Ifnγ* and *Mip-2* expression was only observed in wildtype mice infected with *H. pylori* [161] (Fig. 3a).

Although it was previously hypothesized that loss of SHH expression is mainly caused by the loss of parietal cells, it was further shown that downregulation of SHH expression is associated with parietal cell dysfunction revealing an important role in gastric acid secretion [162] (Fig. 3a). This might be supported by the finding that the *H. pylori*-induced proinflammatory cytokine IL-1β inhibited gastric acid production, intracellular calcium release, and SHH expression in parietal cells via IL-1 receptor signaling leading to gastric atrophy [163] (Fig. 3a). These data underline the importance of the acidic environment in maintaining SHH expression and secretion in the human stomach.

The interference of SHH and gastric acid became more complex when another report indicated that SHH can increase acid secretion by gastric parietal cells through an increase of H^+^/K^+^-ATPase gene expression [164] (Fig. 3a). Phenotypically, transgenic mice that express the hedgehog inhibitor Hhip1 secreted less gastric acid resulting in hypochlorhydria. In these mice, somatostatin was decreased, gastrin gene expression was enhanced and *Shh* mRNA was down-regulated. *Shh* gene expression could be activated through an increase of intracellular calcium, which then activates calcium-specific protein kinase C alpha and beta (PKC-α, PKC-β) (Fig. 3a). Therefore, SHH could function as a ligand that transduces alterations of gastric acidity to the secretion of gastrin by G cells [140].

Overexpression of SHH in human gastric cancers has also been reported [165–167]. In agreement to this, Shh expression can be induced in *H. pylori*-colonized mice in an acid-independent manner [168, 169]. In these mice, *H. pylori* induced infiltration of CD4+ T cells and increased levels of IFNγ and Il-1β in the stomach after six months of infection [169]. Intriguingly, *PC-Shh* ^*KO*^ mice did not develop gastritis in response to *H. pylori* and did not display elevated CD4+ T cells. Macrophages are crucially important in the development of gastritis [42]. In *PC-Shh* ^*KO*^ mice, macrophages were not recruited to the position where ulceration was induced [154]. Interestingly, *H. pylori*-increased SHH mainly occurred in parietal cells of the fundic mucosa and can function as a chemoattractant for macrophages as shown in bone marrow chimera experiments [169] (Fig. 3b). Accordingly, an organoid culture system for the fundic region of the mouse stomach that contained SHH-expressing parietal cells was established to investigate *H. pylori*-mediated SHH signaling. *H. pylori* activated NF-κB, which induced SHH expression in a CagA-dependent manner. Consequently, pharmacological inhibition of NF-κB blocked SHH upregulation [168]. From these data, the authors concluded that SHH acts as a regulator of the initial immune response. Underlining this assumption, CagA-positive *H. pylori* strains were reported to activate SHH expression in the cultured gastric epithelial cell lines AGS, MKN-28, MKN-45 and Kato III cells. Besides SHH, PTCH and GLI were upregulated as well. The authors suggested that *H. pylori* induced NF-κB activity in a CagA-dependent manner to activate SHH expression [170]. However, how CagA is implicated in NF-κB-associated SHH regulation needs to be investigated in more detail, since it is well established that CagA is not directly involved in early *H. pylori*-mediated NF-κB activation [33, 34]. Functionally, expression of SHH in cell culture experiments led to a higher resistance to apoptosis upon infection with *H. pylori* [171], which could explain the hyperproliferative phenotype in response to *H. pylori* infections.

### HH/GLI signaling in the recruitment of bone-marrow derived mesenchymal stem cells (BM-MSCs) to inflamed tissues in response to *H. pylori*

SHH appears to be not only a potential chemoattractant for macrophages, but also for BM-MSCs in chronic inflammation [169, 172]. During chronic infection with *H. pylori*, BM-MSCs are recruited to the site of chronic inflammation to repopulate the gastric epithelium and promote gastric cancer progression [173]. Hence, investigations were performed to elucidate the role of SHH in the regulation of BM-MSCs in the stomach [174]. It was found that IFNγ-induced mesenchymal stem cell (MSC) proliferation required SHH secretion via an autocrine regulatory mechanism. Only MSCs that expressed SHH were finally recruited to the gastric mucosa in response to IFNγ [174] (Fig. 3b). Whether *H. pylori* activated T-lymphocytes produce IFNγ to trigger MSCs in the bone marrow to secrete elevated levels of SHH needs to be investigated in future experiments.

Gastritis can result in MSC proliferation as well. Using a gastrin-deficient mouse model exhibiting a hypochlorhydric phenotype leading to inflammation, parietal cell atrophy and metaplasia, BM-MSCs showed aberrant proliferation and activation of HH/GLI signaling in response to chronic gastric inflammation [175]. Parabiosis experiments demonstrated that circulating signals (e.g. TGFβ) released during *H. pylori*-mediated gastritis induced HH/GLI signaling within bone marrow-derived stromal cells and the rapid recruitment of MSCs to the inflamed stomach [175] (Fig. 3b).

The implication of hedgehog signaling in MSC recruitment is interesting and led to the question about the functional consequences of the recruitment of MSCs to inflamed tissue: tissue regeneration and/or gastric cancer? The finding that *H. pylori* can recruit MSCs that repopulate the epithelium and then transdifferentiate into intraepithelial cancer cells prompted the hypothesis that gastric epithelial cancer can originate from bone marrow-derived cells [173].

## Conclusions

*H. pylori* infections are a paradigm for inflammation-driven cancer. A vast number of reports exist describing the pathophysiological mechanisms, though our knowledge of *H. pylori*-modulated hedgehog signaling in gastric homeostasis and malignant disease is still scarce. At a first glance, the influence of *H. pylori* on SHH expression and function appears controversial. However, upon a closer view on the complex processes it becomes apparently clear that a precise regulation of SHH is a crucial part of gastric physiology. Future studies are necessary to elucidate how gastric HH/GLI signaling is implicated in *H. pylori*-induced pathogenesis as pharmacological targeting of HH/GLI elements represents an attractive approach for the establishment of novel strategies for the treatment of gastric cancer.

## Abbreviations

Abl: Abelson leukemia virus oncoprotein
APC: Adenomatosis polyposis coli
BabA: Blood group antigen-binding adhesin A
BM-MSC: Bone-marrow derived mesenchymal stem cells
BMP: Bone morphogenetic protein
BMP6: Bone morphogenetic protein 6
BOC: Brother of CDO
CAF: Cancer-associated fibroblasts
CagA: Cytotoxin-associated gene A
CagPAI: Cytotoxin-associated gene pathogenicity island
cAMP: Cyclic adenosine monophosphate
CD4+ T cells: CD4 positive T cells
Cdh1: E-cadherin
CDO: Cell adhesion molecule-related/down-regulated by oncogenes
CDX2: Caudal type homeobox 2
CK1: Casein kinase 1
DLG5: Disc large MAGUK scaffold protein 5
ECL: enterochromaffin-like cell
GAS1: Growth arrest specific 1
GKO: Gastrin knock-out
GLI: Glioblastoma-associated-protein
GLI1: Glioblastoma-associated-protein 1
GLI2: Glioblastoma-associated-protein 2
GLI3: Glioblastoma-associated-protein 3
GLIA: Glioblastoma-associated-protein activator form
GLIR: Glioblastoma-associated-protein repressor form
GPR161: G-protein-coupled receptor 161
GSK3β: Glycogen synthase kinase 3 beta
*H. felis*: *Helicobacter felis*
*H. pylori*: *Helicobacter pylori*
H+/K+ ATPase: Proton/Potassium exchanging ATPase
HH: Hedgehog
HHIP: Hedgehog interacting protein
HOXA5: Homeobox A5
IFNγ: Interferon gamma
IFT: Intraflagellar transport
IL10: Interleukin 10
IL11: Interleukin 11
IL12: Interleukin 12
IL1B: Interleukin 1 beta
IL1RN: Interleukin 1 receptor antagonist
IL-8: Interleukin 8
InsGas: Insulin-gastrin
KIF7: Kinesin family member 7
KRAS: Kirsten rat sarcoma viral oncogene homolog proto-oncogene
MALT: Mucosa-associated lymphoid tissue
MIP-2: Macrophage inflammatory protein 2 (aka CXCL2)
MSC: Mesenchymal stem cell
NF-κB: Nuclear factor kappa B
PC-Shh^KO^: Parietal cell-specific Shh knock-out
PKA: Protein kinase A
PKC-α: Protein kinase C alpha
PKC-β: Protein kinase C beta
PTCH: Patched
RT-PCR: Real time PCR
SabA: Sialic acid-binding adhesin A
SHH: Sonic hedgehog
sHip-1: Secreted form of HHIP
SMO: Smoothened
Src: Rous sarcoma oncogene
STAT3: Signal transducer and activator of transcription 3
SUFU: Suppressor of fused
T4SS: Type IV secretion system
TAM: Tumor-associated macrophages
TGFβ: Transforming growth factor beta
TNF-alpha: Tumor necrosis factor alpha
TP53: Tumor protein p53
Tregs: Regulatory T cells
VacA: Vacuolating cytotoxin A
Wnt: Wingless-type MMTV integration site family
WNT2: Wingless-type MMTV integration site family member 2
WNT5A: Wingless-type MMTV integration site family member 5 A
WT: Wildtype
